# Classification of genomic islands using decision trees and their ensemble algorithms

**DOI:** 10.1186/1471-2164-11-S2-S1

**Published:** 2010-11-02

**Authors:** Dongsheng Che, Cory Hockenbury, Robert Marmelstein, Khaled Rasheed

**Affiliations:** 1 Department of Computer Science, East Stroudsburg University, East Stroudsburg, PA 18301, USA; 2 Department of Computer Science, University of Georgia, Athens, GA 30602, USA

## Abstract

**Background:**

Genomic islands (GIs) are clusters of alien genes in some bacterial genomes, but not be seen in the genomes of other strains within the same genus. The detection of GIs is extremely important to the medical and environmental communities. Despite the discovery of the GI associated features, accurate detection of GIs is still far from satisfactory.

**Results:**

In this paper, we combined multiple GI-associated features, and applied and compared various machine learning approaches to evaluate the classification accuracy of GIs datasets on three genera: *Salmonella, Staphylococcus, Streptococcus,* and their mixed dataset of all three genera. The experimental results have shown that, in general, the decision tree approach outperformed better than other machine learning methods according to five performance evaluation metrics. Using J48 decision trees as base classifiers, we further applied four ensemble algorithms, including adaBoost, bagging, multiboost and random forest, on the same datasets. We found that, overall, these ensemble classifiers could improve classification accuracy.

**Conclusions:**

We conclude that decision trees based ensemble algorithms could accurately classify GIs and non-GIs, and recommend the use of these methods for the future GI data analysis. The software package for detecting GIs can be accessed at http://www.esu.edu/cpsc/che_lab/software/GIDetector/.

## Background

Genomic islands (GIs) are clusters of genes in a chromosome that are horizontally transferred from other organisms. Depending on the genetic elements of these genes, GIs can be sub-categorized into (a) pathogenicity islands (PAIs), where genes encode for virulence factors [1]; (b) metabolic islands *(i.e.,* genes encode adaptive metabolic properties) [2]; (c) antibiotic islands (encode antibiotic resistance genes); or (d) secretion islands (encode secretion system genes) [3]. Since different kinds of GIs have different genetic elements, and their sizes might range from 5-500 kilobase pairs, it is a challenging to accurately detect and characterize all GIs in any genome.

With the explosive growth of fully sequenced genomes, the approach of using comparative genomics analysis to detect GIs becomes possible. The comparative genomics approach assumes the availability of at least two or more genomes of related species and strains for any query genome, and it considers the regions with limited phylogenetic distribution in the query genome to be GIs. To our best knowledge, MobilomeFinder [4], MOSAIC [5] and IslandPick [6] use the comparative genomics approach to detect GIs. The major limitation of this approach is that about half of the query genomes do not have minimum number of related species/strains for comparative genome analyses [6]. Thus, detecting GIs in such query genomes may not be applicable. In addition, such methods may also need manual selections of genomes.

An alternative approach of detecting GIs is to use the structural features of GIs. GIs often contain mobile genes such as integrase and transposes. Cheetham and Katz [7] discovered that one PAI in the chromosome of *Dichelobacter nodosus* carries an integrase, which was acquired from *Escherichia coli* phage. GIs are usually flanked by direct repeat (DR) sequences, in which each DR is 16-20 long with nearly perfect sequence repetition, or inverted repeat sequence elements (IS) [8]. In addition, the mobile gene products usually play the roles in inserting and excising of the genomic regions by recombination between the flanking repeats [9]. Another interesting property Hacker and Kaper found is that 75% of the insertion sites of GIs are at the 3′-end of a transfer RNAs (tRNAs) [2].

Another interesting feature that can tells GIs from non-GIs is based on the sequence composition of the genome. Typically, each genome generally has its own unique sequence composition signature, and thus the sequence compositions between GIs, which are from an alien genome, and the rest of the host genome are different. For instance, the measurement of guanine and cytosine (G+C) contents in a chromosome showed that 20-30% genomic regions carried atypical G+C contents which were possibly GI-associated [10]. The combination of codon bias and Codon Adaption Index (CAI) was used to detect alien genomic regions [11,12]. Besides, Karlin [13] used dinucleotide frequency difference (δ* difference) to identify possible GIs. In order to improve the discrimination power for detecting alien gene clusters, Tsirigos and Rigoutsos [14] extended the 2 mers (i.e., δ* difference) to 8-9 mers. Recently, Vernikos and Parkhill [18] proposed a new model, interpolated variable order motifs, to detect horizontally acquired genes. This new method overcomes the low discrimination power problem using the lower-order motif models, and the extremely low frequency problem of observed motifs using the higher-order motif models.

In order to improve the detection power, the integration of multiple GI-associated features for detecting GIs may be applied. IslandPath [16] is a web-server that displays the G+C contents of open reading frames (ORFs), δ* difference (dinucleotide), the location of mobile genes, and the location of tRNAs. IslandPath leaves users to judge whether a genomic region are GIs or not, based on provided multiple feature values. A recent study on IslandPath has shown that using the feature of δ* difference only leads to the low specificity problem, while using the combined features of δ* difference and mobile gene leads to the low sensitivity problem [6]. Garcia-Vallve et al. [17] used a simple rule-based algorithm to identify horizontally acquired gene cluster. The gene cluster is considered to be horizontally acquired if either the G+C content and codon usage deviate by more than 1.5 standard deviations from the mean values, or the G+C content is extremely high or low. Recently, Vernikos and Parkhill [18] combined multiple GI-associated features such as sequence composition and mobile gene, and used Relevance Vector Machine (RVM), a model similar to Support Vector Machines (SVMs) but exploiting fewer basis functions, to classify GIs. While multiple features have been used in a few studies previously, comprehensive machine learning approaches and performance comparison have not been systematically studied, leaving room for improvement for predicting GIs.

In this paper, we present our work about classifying several genomic island datasets using supervised machine learning algorithms, and show that decision tree method perform better than other machine learning models including naive Bayesian, Bayesian networks, neural networks, simple logistic and support vector machines (SVMs) in general. We will show decision tree based ensemble algorithms can further improve classification accuracy by up to 5.9%.

## Results and discussion

### Feature analysis

In this study, we used the datasets of GIs and non-GIs from three genera: *Salmonella*, *Staphylococcus*, and *Streptococcus.* For each instance (either GI or non-GI) of these datasets, eight feature values, *i.e.,* Interpolated Variable Order Motif (IVOM), Insert point, Size, Density, Repeats, Integrase, Phage and RNA, were obtained. The description summary of the eight features is listed in Table 1 (See Methods for more details).

**Table 1:** The descriptions of the features associated with genomic islands

Feature	Description
IVOM	Interpolated Variable Order Motif compositional score (Relative Entropy)
Insertion Point (IP)	Binary: “1” if within a CDS locus, “0” otherwise
Size	Size of each genomic region in bp
Density	Number of genes per kb
Repeats	Binary: “1” if repeats present, “0” otherwise
Integrase	Binary: “1” if containing integrase-like protein domain, “0” otherwise
Phage	Binary: “1” if containing phage-related protein domain, “0” otherwise
RNA	Binary: “1” if containing non-coding RNA, “0” otherwise

In order to evaluate each of eight features, we define the signal to noise ratio (G2N) as the distance of the arithmetic means of the GI and non-GI classes divided by the sum of the corresponding standard deviations, *i.e.,*

     (1)

where *μ_GI_* and *μ_non_GI_* are the mean feature values from the GI dataset and non-GI dataset, respectively. *σ_GI_* and *σ_non_GI_* are their standard deviations from the GI dataset and non-GI dataset.

We analyzed the feature analyses for the genera of *Salmonella, Staphylococcus, Streptococcus* and their all mixed-up datasets. The evaluation of the eight features on these four datasets shows that Integrase, Phage and Repeats are the most informative features. This can be easily to see in the datasets of *Streptococcus,* where the G2N values of Integrase, Repeats and Phages are 1.02, 0.94 and 0.82 respectively (See Table 2). The effectiveness of these features in both individual genera and their mixed-up datasets strongly suggests the existence of mobile elements and flanking repeats in all GI families (Table 2 and Additional file 1).

**Table 2:** Feature quality analysis on dataset of Streptococcus

Feature	G2N	*µ_GI_*	*σ_GI_*	*µ_non_GI_*	*σ_non_GI_*
IVOM	0.51	21.06	12.97	9.96	8.73
IP	0.89	0.44	0.50	0	0
Size	0.06	19879	16390	17811	15890
Density	0.26	1.15	0.40	0.99	0.20
Repeats	0.94	0.74	0.44	0.08	0.27
Integrase	1.02	0.65	0.48	0.02	0.14
Phage	0.82	0.41	0.50	0	0
RNA	0.23	0.17	0.38	0.04	0.19

The effectiveness of some features is genus-specific. For instance, the feature of Insertion point in the CDS is very informative in the genera of *Streptococcus* and *Staphylococcus,* but not in *Salmonella.* The feature of RNA is also genomic-specific. Interestingly, unlike the feature of Insertion point, RNA is informative for the genus of *Salmonella,* with small contribution for the genera of *Streptococcus* and *Staphylococcus.* The feature of Density seems to be uninformative to the genus of *Salmonella,* but is informative to the genus of *Staphylococcus.*

Out of eight features analyzed, the feature ‘Size’ is the least informative. This can be explained by the random sampling process of non-GI datasets, whose genomic region size distribution was roughly the same as that of GIs. However, in many cases, an uninformative single feature does not imply that that feature will not contribute to the whole model when multiple features are applied. Previous studies have shown that the ‘Size’ feature was indeed contributive in the RVM model [18].

### Decision tree approaches outperform other machine learning algorithms

**Figure 1:**
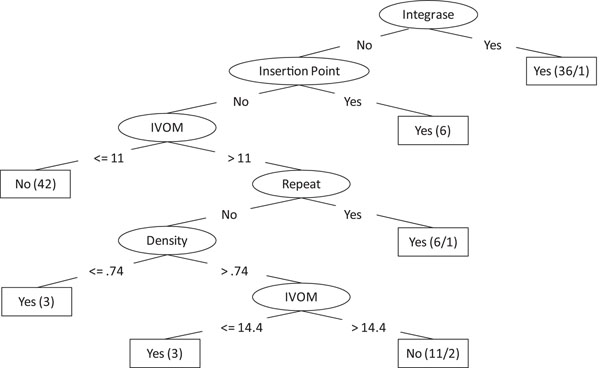
**Decision tree model** An example of a decision tree model for the genomic island classification of *Streptcoccus.* Each interior node is one of the features (i.e.,Density, Insertion point, Integrase, IVOM, and Repeat), while each leaf node is the classification (i.e., “Yes” indicates the test sequence segment is GI, while “No” indicates it is a nonGI).

Decision tree classification is one of most widely used machine learning methods. A decision tree classification model is represented by a tree-like structure, where each internal node represents a test of feature, with each branch representing one of the possible test results, and each leaf node represents the classification. Due to the explosive growth of biological data in the past decade, the decision tree approach has many successful biological applications, including coding and noncoding DNA classification [19], protein secondary structure prediction [20], and operon structure classification [21]. In this study, we used two decision tree methods, Classification and Regression Tree (CART) and J48 (an extended Java implementation version of C4.5 algorithm), for the GI classification. Figure 1 demonstrates a J48 decision tree model built based on the dataset of the genus of *Streptococcus.*

For performance comparision, we also used other five machine learning algorithms, including naive Bayesian, Bayesian networks, logistic regression, neural network, and SVMs. We used the WEKA machine learning package [22] because all these algorithms have been implemented in the package. For the classification of each algorithm, default parameters provided in the package were used.

Table 3 lists five performance evaluation metrics (see Methods for more details) of each algorithm on the mixed-up dataset of three genera. As we can see from Table 3, the J48 decision tree approach has the highest sensitivity value (0.858), though its specificity is a little bit lower than the other five algorithms. Since F-Measure, accuracy, and AUC take both sensitivity and specificity into consideration, they reflect the performance more accurately than sensitivity and specificity do separately. The J48 decision tree approach unanimously showed the best performance using the metrics of F-Measure, accuracy and AUC. Another decision tree approach, CART is ranked to the second best classifier.

**Table 3:** Performance comparison among machine learning algorithms

Method	Sensitivity	Specificity	F-Measure	Accuracy	AUC
CART	0.813	0.807	0.811	0.810	0.855
Naive Bayesian	0.589	0.893	0.710	0.743	0.811
BayesianNet	0.604	0.914	0.727	0.760	0.839
J48	0.858	0.843	0.850	0.850	0.892
Logistic	0.628	0.893	0.738	0.762	0.845
Neural Network	0.659	0.849	0.742	0.754	0.833
SVM	0.565	0.902	0.695	0.735	0.734

### Ensemble learning algorithms can improve classification accuracy

Using the J48 decision tree approach as the baseline for classification, we applied four decision-tree-based ensembles, adaBoost, bagging, MultiBoost, and random forest in classifying four datasets *(Salmonella, Staphylococcus, Streptococcus* and their mixed-up dataset). Again the WEKA package and default parameters were used. Table 4 lists five performance evaluation metrics of each ensemble algorithm on the mixed-up dataset. We found that, in general, all ensemble algorithms could improve classification.

**Table 4:** Performance comparison between J48 and decision tree based ensembles

Method	Sensitivity	Specificity	F-Measure	Accuracy	AUC
J48	0.858	0.843	0.850	0.850	0.892
AdaBoost	0.890	0.910	0.902	0.900	0.932
Bagging	0.870	0.872	0.873	0.871	0.940
MultiBoost	0.880	0.871	0.876	0.876	0.942
Random Forest	0.819	0.889	0.859	0.850	0.908

The ensemble classifiers include bagging, AdaBoost, MultiBoost, random forest.

**Figure 2:**
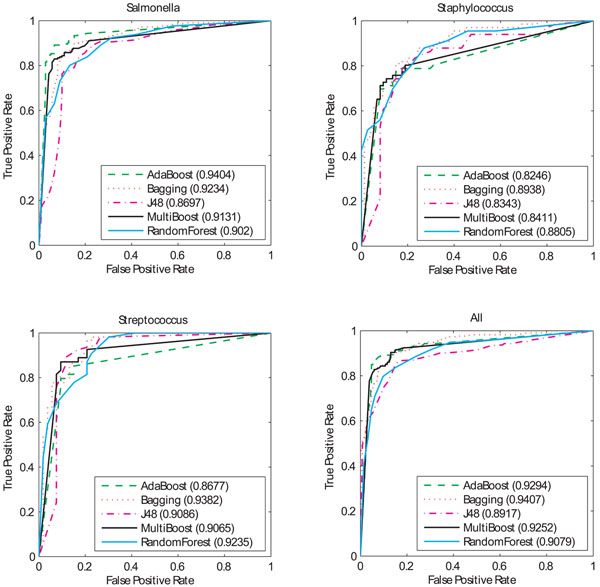
**ROC curves of decision tree based ensembles** For each dataset of *Streptococcus, Staphylococcus*, *Salmonella,* and all-three. ROC curves of adaBoost, bagging, multiBoost, random forest, and single J48 are calculated.

For the visualization purpose, we also provide ROC curves and their corresponding AUC values of four ensemble algorithms, as well as the J48 algorithm, on each of four datasets (See Figure 2). Bagging has the highest AUC values based on the evaluation of four datasets. Compared to those of a single J48 algorithm, the AUC values of the bagging algorithm are 5% (0.92 versus 0.87), 6% (0.89 versus 0.83), 4% (0.94 versus 0.90), and 5% (0.94 versus 0.89) higher in the models of *Salmonella, Staphylococcus, Streptococcus,* and mixed-up three, respectively. It is interesting to see that adaBoost performs the best among all algorithms in the dataset of *Salmonella*, but no improvements in the datasets of *Staphylococcus* and *Streptococcus*.

Since one genome may contain several or up to dozens of GIs [6,15], we may unavoidably face the problem of collecting small datasets for GI classification. The datasets used in our study were from thirty-seven strains of three different genera, with 331 GIs and 337 non-GIs. The relative small datasets for the genera of *Staphylococcus* and *Streptococcus* led to the unsmooth ROC curves, as shown in Figure 2. As more genomes of the strains in these genera will be sequenced and the training datasets become bigger, the ROC curves will be more accurately reflect our classification algorithms.

**Figure 3:**
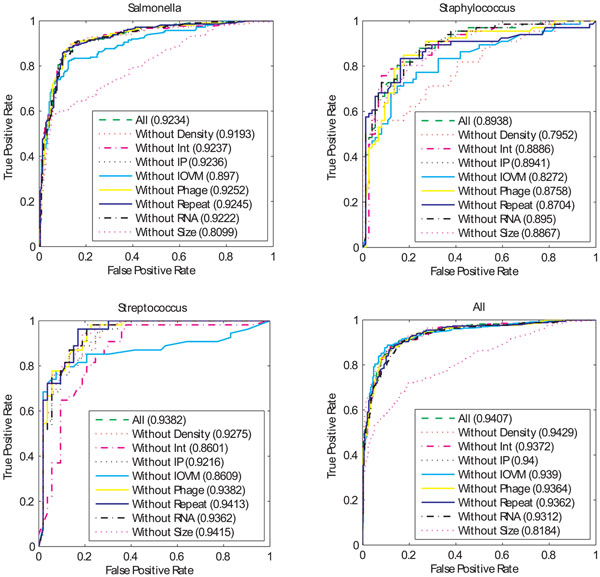
**ROC curves for the “leave-one-feature-out” models using bagging** For each dataset of *Streptococcus, Staphylococcus, Salmonella,* and all-three. ROC curves of the “leave-one-feature-out” models are calculated.

We further investigated the contribution of each single feature by using “leave-one-feature-out” model, where in each experiment one feature was removed from all feature model. Figure 3 lists the ROC curves and their corresponding AUC values of the bagging algorithm on each of four datasets (See Additional file 2 for the corresponding one of adaBoost). By analyzing each feature using the “leave-one-feature-out” model, We found that the feature “Size” is very informative in the dataset of *Salmonella* (Figure 3), where we can see that the classification power dropped by 11.3% of the AUC value when the Size feature was removed. The high contribution of the Size feature in the model of *Salmonella* suggests that other GI-associated features do correlate with the Size feature. Another observation from these classification results is that a single feature can be dropped from the multiple features in many cases, without affecting the classification performance dramatically. For instance, the AUC values are 0.924, 0.924, 0.925 and 0.924 for the models without the feature of Integrase, Insertion point, Phage, and Repeat, while the AUV value for all feature model is 0.923. This analysis indicates the contributions of some feature values are redundant, even they are informative by applying the signal-to-noise feature analysis.

Overall, when multiple features are considered together, the most informative features are the Size and IVOM features. This is in contrast with the signal-to-noise analysis, where the features of Integrase, Phage and Repeats are most informative. These results bring us an attention that the feature selection should be taken cautiously when applying the single feature analysis.

### Comparison to other approaches

We compared the classification results of our J48-based bagging algorithm with a sequence composition based approach, AlienHunter [15]. We found the overall accuracy of our approach on the genera of *Salmonella, Staphylococcus* and *Streptococcus* are 14% (0.87 versus 0.73), 8% (0.79 versus 0.71), 8% (0.86 versus 0.78) higher than that of AlienHunter, respectively. The superior performance of our approach to AlienHunter is that our approach uses multiple features, while AlienHunter uses the information of sequence composition only.

We also compared the bagging with the classification results of the RVM method in a previous study [15], and found that the AUC values of the bagging algorithm are 9% (0.92 versus 0.83), 7% (0.89 versus 0.82), and 10% (0.94 versus 0.84) higher in the models of *Salmonella, Staphylococcus,* and mixed-up three, respectively, and is same (0.94 versus 0.94) in the model of *Streptococcus.*

### Application of trained models for the whole-genome scale GI detection

The trained models based on J48 decision tree based ensemble algorithms can be used to detect the whole genome scale GIs of prokaryotic genomes. We have developed an automated software package that contains the functionalities of downloading genome data, extracting GI associated feature values, and predicting GIs based on our trained models from this study. The software package was written in C# and tested on Windows, and it is available at http://www.esu.edu/cpsc/che_lab/software/GIDetector/. The detailed description, as well as the usage, of our software package will be addressed elsewhere (in preparation).

Currently, the software contains models for the genera of *Salmonella, Staphylococcus, Streptococcus.* Thus, it is advisable to use the models of the same genus to detect any genome belonging to these three genera. For the GI detection of the other species, we provide a general model which was trained on three mixed datasets. The low decreasing of classification accuracy on the tests of all mixed datasets suggests that the models of different genera share a super GI structure model, which is fairly applicable to any other genomes. With more sequenced genomes available, we believe that more GIs will be studied, confirmed and assembled. To this end, our software package also provides a platform for users to build GI models for their own needs.

## Conclusions

In this paper, we have presented a comparative study applying several machine learning algorithms for classifying the genomic island dataset. Our experimental results have shown that the J48 decision tree approach performed very well based on multiple performance evaluation metrics. Furthermore, decision tree based ensemble algorithms were shown to improve the performance over the single decision tree algorithm. These results suggest that such decision tree based ensembles can be applied for genomic island classification.

The analyses of the contribution of single features by using signal-to-noise analysis and leave-one-feature-out analysis, suggests that feature interaction is quite complicated in this domain. While the single feature analysis sheds new lights on the utility of each feature, it does not tell us that it will be informative or redundant when multiple feature models are integrated.

## Methods

### GI and non-GI Datasets

We obtained the datasets of GIs (positives) and non-GIs (negatives) of genus *Salmonella, Staphylococcus,* and *Streptococcus* processed by Vernikos and Parkhill [18]. The detection of GIs was based on the fact that a genomic region limited in one lineage was more likely to have been horizontally acquired than to have been deleted independently from multiple lineages [23]. On the other hand, if a genomic region that is present in one lineage and most of strains of another lineage, we consider the genomic region that is missing in some strain of another lineage is involved in the deletion, rather than horizontally acquired. Based on these studies, putative GIs could be derived by combining comparative analysis and the maximum parsimony models [24]. The numbers of GIs selected from the genus *Salmonella, Staphylococcus* and *Streptococcus* were 211, 54 and 66, respectively, with the total positives of 331.

For each detected GI of a genome, a corresponding non-GI with the same genomic region size was randomly sampled within the inter-GI regions. The redundant non-GIs that sampled from the different strains of the same genus were removed, and the sampling results of non-GIs for genus *Salmonella Staphylococcus* and *Streptococcus* were 210, 53 and 74, respectively, with the total negatives of 337.

For each instance (either GI or non-GI) of these datasets, eight feature values, *i.e.,* IVOM, Insert point, Size, Density, Repeats, Integrase, Phage and RNA, were obtained. The Interpolated Variable Order Motif (IVOM) score measures the composition bias of a genomic region relative to the background genomic region by using the relative entropy of both low and high order motifs in a genomic region over the background genome [15]. Repeats were detected by the REPuter [25] program which starts with finding exact repeats, and then significantly degenerated repeats to allow mismatch, insertion, or deletion. The protein domains associated with integrases and phages were retrieved from the Pfam protein families database [26]. Finally, RNA regions was detected by tRNAscan-SE [27].

### Problem formulation

The goal of this study is to construct classifiers that can accurately classify the gnomic islands and non-genomic islands from the genomic data in prokaryotic organisms. The classifiers can then be used as the basis for classifying any genomic segment in a whole genome. This is a classical supervised learning problem that applies a learning algorithm on the training data and performs prediction on the test data. The training examples are a set of tuples <*x, c*>, where *c* is the class label (i.e., either genomic island (GI) or non-genomic island), and *x* is the set of attributes for the instances. In this study, eight attributes (IVOM, Insert point, Size, Density, Repeats, Integrase, Phage and RNA) are included. The learning algorithm is trained on the positive *E*+ (i.e., GIs) and negative E- (i.e., non-GIs) examples to construct a classifier *C*(*x*) that distinguishes between these examples.

### Decision tree and decision tree based ensemble algorithms

#### Decision tree

In this study, we use J48 in WEKA, the Java implementation of C4.8 algorithm. C4.8 is the latest research version for the C4.5 algorithm, which is one of the best-known and most widely-used decision tree algorithms. C4.5 extends the ID3 algorithm by addressing several important issues. The C4.5 algorithm handles numerical attributes and missing values, it also incorporates post-pruning process to handle the over-fitting problem.

The ID3 algorithm [28] implements a top-down greedy search schema to search through all possible tree spaces. It starts with all training set (*S*) and chooses the best feature as the root node. The best feature should have the highest calculated value of *information gain (IG*), which is defined as

     (2)

where *E*(*S*) is the entropy of *S*, which in turn is defined as

*E*(*S*) = –*p_GI_log*_2_*p_GI_* – *p_NG_log*_2_*p_NG_*     (3)

where *p_GI_* is the probability that the selected dataset is of GI *(i.e.,* the percentage of GIs in *S*), and conversely, *p_NG_* is for non-GI. *Value(A)* is the set of all possible values for the feature A, one of eight features in this study. *S_v_* is the subset of *S* for which feature *A* has the value of *v (i.e., S_v_* = {*s* ∈ *S*|*A*(*s*) = *v*}).

The ID3 algorithm splits the set based on the possible values of the selected best feature. If the all instances in a subset have the same output value *(i.e.,* either GI or non-GI), then the process stops for that branch, and that node is a terminal node. If the subset does contain instances from two classes (both GI and non-GI), this process will be repeated until there are no further distinguishing features can be determined.

#### Bagging

Bootstrap Aggregating [29], better known as bagging, is a method whose classification takes the majority votes of multiple classifiers thus forming a hypothetical “committee”. Each classifier in the committee is un-weighted and each classifier is a decision tree model in this study. Each decision tree classifier model is trained on a subset of the initial training set. The training set of each classifier model can be sampled by bootstrap sampling, *i.e.,* randomly selecting a subset of given dataset with replacement, allowing for sample values to be independent of one another.

#### AdaBoost

Adaptive boosting (adaBoosting) [30] uses the weighted data sampling and voting scheme. The algorithm starts by building the first base classifier, which is trained on the dataset with equal weights. For the construction of subsequent classifiers, the instances misclassified by the previous classifier are assigned higher weights, while the weights of the instances that are correctly classified remain the same. The weights of all instances in the whole dataset are then normalized so that all weights add up to 1, and then used for sampling for the next classifier. The final classification for an instance is based on the classifications by all classifiers, with each classifier weighted also. The class with the highest weighted votes is the final classification.

#### MultiBoost

In the MultiBoost algorithm [31], the classification is the weighted aggregate of multiple committees, rather than un-weighted aggregating as in bagging. Furthermore, each committee of MultiBoost itself is a decision tree ensemble known as AdaBoost [32]. AdaBoost is a weighted aggregating of committees, where each committee member is a basic decision tree model. Each decision tree classifier is constructed on a subset of given dataset. The sampling of the subset of dataset is a random sampling with replacement for the first classifier construction. In later classifier construction, however, the instances misclassified in previous classifiers are assigned high weights. The detailed algorithm and the determination of weights can be referred to in [32].

#### Random forest

Random forest [33] is similar to bagging in that both use the bootstrap sampling technique to select the subset of the base training dataset to train decision tree models, and both use the un-weighted aggregating of committees for the final classification. However, the selection of the best feature in the process of the decision tree structure is different. In the random forest approach, *m* features out of *M* features are randomly selected, and the optimal value of *m* is usually the square root of *M.* The best feature out of *m* features is determined based on the calculated information gain. In addition, each tree is fully grown and not pruned in random forest.

### Performance evaluation

A ten-fold cross-validation scheme is used to evaluate the classification accuracy of all classifiers. In particular, the known GI and non-GI datasets are evenly separated into ten parts, and the first part is evaluated based on the model trained from the remaining nine parts. This process continues until all ten parts have been evaluated. The overall performance metric is the average of all ten separate evaluations. True positives (TP) are the number of GIs predicted to be GIs. False negatives (FN) are the number of GIs predicted to be non-GIs. True Negatives (TN) are the number of non-GIs predicted to be non-GIs. False positives (FP) are the number of non-GIs predicted to be GIs. We focus on the following validation measures:

*Sensitivity* = *TP*/(*TP* + *FN*)     (4)

*Specificity* = *TN*/(*TN* + *FP*)     (5)

*Accuracy* = (*TP* + *TN*)/(*TP* + *TN* + *FN* + *FP*)     (6)

*F* – *measure* = *2* * *Sen* * *Spec*/(*Sen* + *Spec*)     (7)

We have also used the area under the ROC (Receiver Operating Characteristic) curve (AUC) to measure the classification performance. The AUC value is the percentage of correctly classified one pair of samples, with each from one class. The AUC takes the value between 0 and 1, and a random classifier has the AUC value of 0.5. Theoretically, a well-performing classifier should have a high AUC value.

## Competing interests

The authors declare that they have no competing interests.

## Authors' contributions

DC, RM, and KR conceived the project and designed the experiments; DC conduct part of the experiments and wrote the manuscript; CH conducted part of the experiments and developed the software package; All authors read and approved the final manuscript.

## Supplementary Material

Additional file 1 **Feature quality analysis.** This file contains feature analysis on three datasets, *Staphylococcus*, *Salmonella*, and all-three mixed. Click here for file

Additional file 2 ROC curves for the “leave-one-feature-out” models using adaBoost. Click here for file
