# Supplementary material for: Classification of genomic islands using decision trees and their ensemble algorithms
Source: BMC Genomics. 2010 Nov 2;11(Suppl 2):S1. doi: 10.1186/1471-2164-11-S2-S1 (PMC2975412; doi:10.1186/1471-2164-11-S2-S1)
Supplement: Additional file 1 — Feature quality analysis. This file contains feature analysis on three datasets, Staphylococcus, Salmonella, and all-three mixed. [file 1471-2164-11-S2-S1-S1.pdf]

Additional file 1:

TABLE I
Feature quality (*Salmonella*)

Salmonella					
#	G2N	μ_{GI}	σ_{GI}	$\mu_{non\text{-}GI}$	$\sigma_{non\text{-}GI}$
f_1	0.45	13.98	14.02	4.85	6.32
f_2	0.10	0.01	0.10	0	0
f_3	0.05	12964.35	15855.83	14783.85	19354.48
f_4	0.16	1.02	0.38	0.92	0.24
f_5	0.37	0.26	0.44	0.03	0.18
f_6	0.47	0.28	0.45	0.01	0.12
f_7	0.45	0.27	0.44	0.01	0.12
f_8	0.43	0.27	0.44	0.02	0.14

TABLE II:
Feature quality (*Staphylococcus*)

Staphylococcus					
#	G2N	μ_{GI}	σ_{GI}	$\mu_{non\text{-}GI}$	$\sigma_{non\text{-}GI}$
f_1	0.10	8.96	5.38	7.39	11.04
f_2	0.51	0.21	0.41	0	0
f_3	0.10	20266.18	20383.67	25432.43	29523.19
f_4	0.43	1.19	0.33	0.92	0.30
f_5	0.56	0.56	0.50	0.11	0.31
f_6	0.65	0.56	0.50	0.07	0.25
f_7	0.69	0.44	0.50	0.01	0.12
f_8	0.19	0.17	0.38	0.05	0.23

TABLE III:
Feature quality (All 3)

All					
#	G2N	μ_{GI}	σ_{GI}	$\mu_{non\text{-}GI}$	$\sigma_{non\text{-}GI}$
f_1	0.37	14.14	13.08	6.21	8.17
f_2	0.37	0.12	0.33	0	0
f_3	0.05	15548.32	17226.70	17598.24	21907.29
f_4	0.23	1.07	0.38	0.93	0.25
f_5	0.47	0.40	0.49	0.06	0.23
f_6	0.57	0.40	0.49	0.03	0.16
f_7	0.54	0.32	0.47	0.01	0.11
f_8	0.34	0.23	0.42	0.03	0.17
