# Supplementary figures and images for: Classification of genomic islands using decision trees and their ensemble algorithms
Source: BMC Genomics. 2010 Nov 2;11(Suppl 2):S1. doi: 10.1186/1471-2164-11-S2-S1 (PMC2975412; doi:10.1186/1471-2164-11-S2-S1)

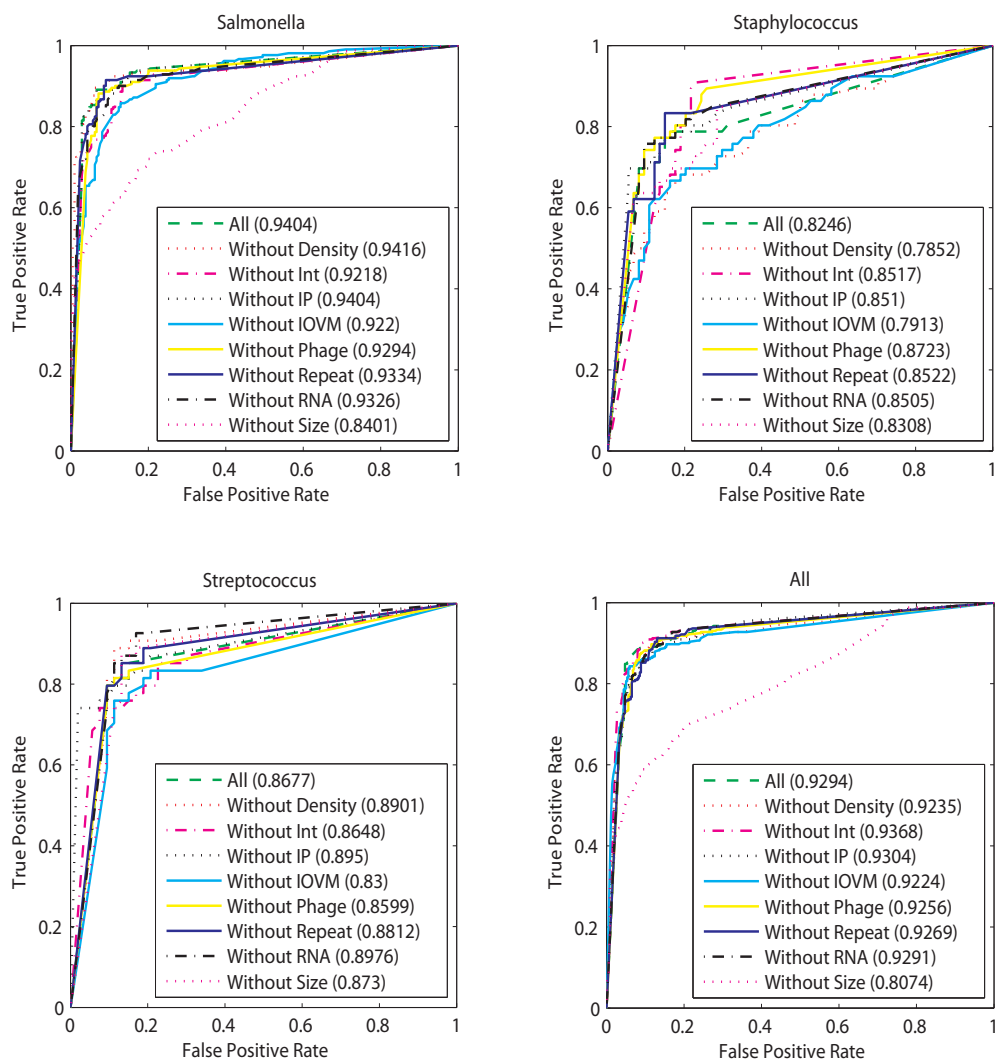

Fig. 1: ROC curves for the “leave-one-feature-out” models using adaboost.

Supplement: Additional file 2 — ROC curves for the “leave-one-feature-out” models using adaBoost. [file 1471-2164-11-S2-S1-S2.pdf]
